# 1-[6-(9*H*-Carbazol-9-yl)hex­yl]-2-phenyl-1*H*-benzimidazole

**DOI:** 10.1107/S1600536809046820

**Published:** 2009-11-14

**Authors:** Yu-Ling Zhao, Tian-Zhi Yu, Jing Meng

**Affiliations:** a School of Chemical and Biological Engineering, Lanzhou Jiaotong University, Lanzhou 730070, People’s Republic of China; b Key Laboratory of Opto-Electronic Technology and Intelligent Control, (Lanzhou Jiaotong University), Ministry of Education, Lanzhou 730070, People’s Republic of China

## Abstract

The mol­ecule of the title compound, C_31_H_29_N_3_, contains a hexyl chain, a coordination unit (benzimidazole) and a functional group (carbazole). The benzimidazole ring is not coplanar with either the phenyl ring or the carbazole system, making dihedral angles of 43.26 (3) and 39.03 (2)°, respectively. The dihedral angle between the phenyl ring and the carbazole system is 24.42 (3)°. The hexyl C_β_ atom (with respect to benzimidazole) deviates by 1.124 (2) Å from the benzimidazole plane, although the C_α_ atom lies in the plane. The hexyl C_β_ atom (with respect to carbazole) deviates by 1.315 (1) Å from the carbazole plane, although the C_α_ atom lies in the plane. The crystal structure is stabilized by inter­molecular C—H⋯π inter­actions.

## Related literature

For applications of benzimidazole-containing compounds as human cytomegalovirus inhibitors and anthelmintic agents, see: Spasov *et al.* (1999 ▶); Zhu *et al.* (2000 ▶). Benzimidazole derivatives can act as ligands to transition metals for modeling biological systems, see: Bouwman *et al.* (1990 ▶) and for organic light-emitting devices (OLEDs), see: Huang *et al.* (2004 ▶); Si *et al.* (2007 ▶). For bond-length data, see: Allen *et al.* (1987 ▶).
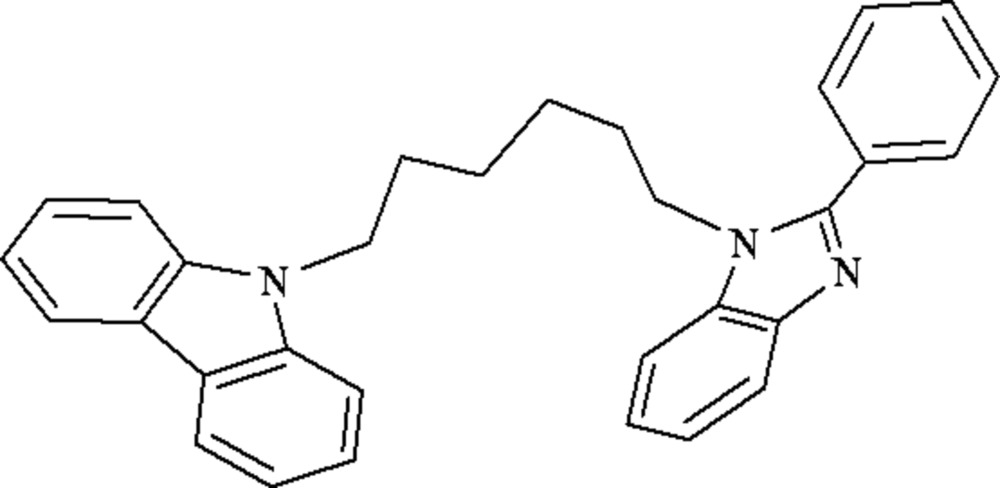


## Experimental

### 

#### Crystal data

C_31_H_29_N_3_
*M* *_r_* = 443.57
Monoclinic, 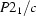

*a* = 8.6623 (6) Å
*b* = 31.582 (2) Å
*c* = 8.9187 (6) Å
β = 96.3120 (10)°
*V* = 2425.1 (3) Å^3^
*Z* = 4
Mo *K*α radiation
μ = 0.07 mm^−1^
*T* = 293 K
0.43 × 0.18 × 0.12 mm

#### Data collection

Bruker SMART APEX CCD area-detector diffractometer
Absorption correction: multi-scan (*SADABS*; Sheldrick, 1996 ▶) *T*_min_ = 0.970, *T*_max_ = 0.991
13496 measured reflections
4757 independent reflections
3374 reflections with *I* > 2σ(*I*)
*R*_int_ = 0.026

#### Refinement

*R*[*F* ^2^ > 2σ(*F* ^2^)] = 0.046
*wR*(*F* ^2^) = 0.119
*S* = 1.01
4757 reflections
307 parameters
H-atom parameters constrained
Δρ_max_ = 0.12 e Å^−3^
Δρ_min_ = −0.14 e Å^−3^

### 

Data collection: *SMART* (Siemens, 1996 ▶); cell refinement: *SAINT* (Siemens, 1996 ▶); data reduction: *SAINT*; program(s) used to solve structure: *SHELXS97* (Sheldrick, 2008 ▶); program(s) used to refine structure: *SHELXL97* (Sheldrick, 2008 ▶); molecular graphics: *SHELXTL* (Sheldrick, 2008 ▶); software used to prepare material for publication: *SHELXTL*.

## Supplementary Material

Crystal structure: contains datablocks global, I. DOI: 10.1107/S1600536809046820/jh2111sup1.cif

Structure factors: contains datablocks I. DOI: 10.1107/S1600536809046820/jh2111Isup2.hkl

Additional supplementary materials:  crystallographic information; 3D view; checkCIF report

## Figures and Tables

**Table 1:** Hydrogen-bond geometry (Å, °)

*D*—H⋯*A*	*D*—H	H⋯*A*	*D*⋯*A*	*D*—H⋯*A*
C28—H28⋯*Cg*1	0.93	2.78	3.665 (2)	159
C18—H18*A*⋯*Cg*2	0.97	2.87	3.596 (3)	133

*Cg*1 and *Cg*2 are the centroids of the 13-atom carbazole ring and the C19/C24/C25/N2/N3 imidazole ring, respectively.

**Fig. 1.**
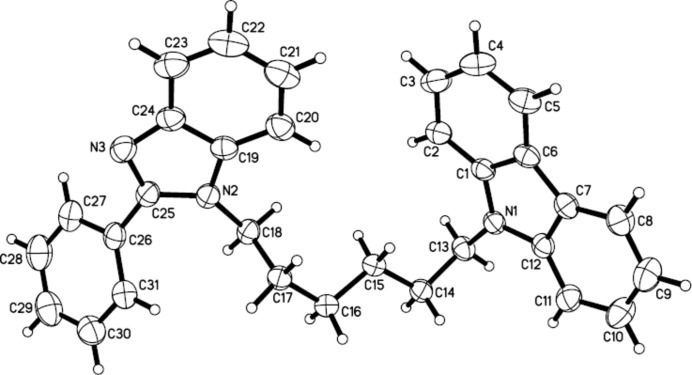
The molecule structure of the title compound with the atom numbering scheme. Displacement ellipsoids for non-hydrogen atoms are drawn at the 30% probability level.

**Fig. 2.**
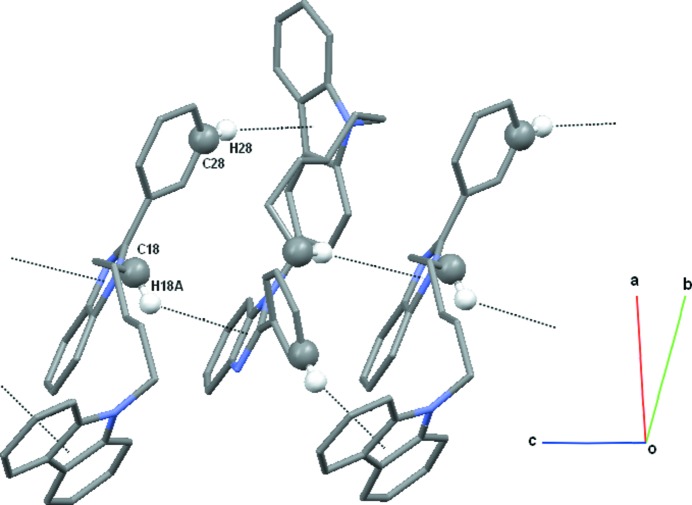
Packing diagram of the title compound, showing C—H···π stacking interactions as thin black lines. Colour code: grey: C; white: H. H atoms not involved in hydrogen bonding have been omitted for clarity.

## supplementary crystallographic information

### Comment

The benzimidazole moiety is an important heterocyclic nucleus which has been used extensively in medicinal chemistry. Many benzimidazole-containing compounds have found commercial applications in human cytomegalovirus inhibitor and anthelmintic agents (Zhu *et al.*, 2000; Spasov *et al.*, 1999). Moreover, benzimidazole derivaterives can act as ligands to transition metals for modeling biological systems (Bouwman *et al.*,1990) and for organic light-emitting devices (OLEDs) (Huang *et al.*, 2004; Si *et al.*, 2007). 2-Phenyl-benzimidazole-based cyclometalated iridium complexes are excellent phosphorescence materials and the devices using these complexes as dopants exhibit very high efficiencies (Huang *et al.*, 2004). Recently we modified 2-phenyl-benzimidazole with multifunctional charge-transporting groups by nonconjugated aliphatic linkage to improve the charge-transporting characteristic of 2-phenyl-benzimidazole-based cyclometalated iridium complexes. In this paper, we report the crystal structure of a new ligand containing hole-transporting carbazole group, 1-(6-carbazolylhexyl)-2-phenyl-benzimidazole.

The molecular structure of the title compound and the *ORTEP* structure is shown in Fig. 1. The bond lengths and angles in the molecule are within normal ranges (Allen *et al.*, 1987). The benzimidazole ring and the phenyl ring as well as the benzimidazole ring and the carbazole ring are not coplanar, making the dihedral angle of 43.26 (3)° and 39.03 (2)°, respectively. The dihedral angle between the phenyl ring and the carbazole ring is 24.42 (3)°. The C17 atom deviates by 1.124 (2) Å from the benzimidazole plane, although the C18 atom lies in the plane. The C14 atom deviates by 1.315 (1) Å from the carbazole plane, although the C13 lies in the plane. The torsion angles of C14—C13—N1—C1, C14—C13—N1—C12, C17—C18—N2—C19 and C17—C18—N2—C25 are -98.15 (2)°, 83.41 (3)°, 79.54 (1)° and -111.44 (2)°, respectively.

The crystal structure is stabilized by intermolecular C—H···π interactions [*Cg*1 and *Cg*2 are the centroids of 13 atoms carbazole ring and the C19C24C25/N2N3 imidazole ring, respectively.] (Table 1, Fig. 2).

### Experimental

1-(6-carbazolylhexyl)-2-phenyl-benzimidazole was obtained in three steps. Firstly, 2-phenyl-benzimidazole was synthesized by reacting *o*-phenylendiamine and benzoic acid in presence of polyphosphoric acid under N_2_ at 433 K for 8 h. Secondly, 9-(6-bromohexyl)-carbazole was prepared by reacting cabazole and 1,6-dibromohexane in the mixed solvent of toluene and aqueous 50% sodium hydroxide, in which tetrabutyl ammonium bromide was used as the phase-transfer catalyst. Finally, under N_2_ solid NaH and 2-phenyl-benzoimidazole in anhydrous DMF was stirred at 353 K for 2 h, then 9-(6-bromohexyl)-carbazole was added. The mixed solution was stirred overnight at room temperature. The crude product was chromatographed using ethyl acetate / hexane (1:2, V/V) to afford the title compound. Yield, 75.12%. m. p. 403–405 K. ^1^H-NMR (500 MHz, CDCl_3_): 8.09(d, 2H), 7.81(d, 1H), 7.64 (s, 2H), 7.46(m, 5H), 7.31(d, 4H), 7.23 (m, 3H), 4.21–4.13 (m, 4H,), 1.78–1.70 (m, 4H), 1.21–1.19 (m, 4H).

Yellow tabular single crystals of the title compound were obtained by slow evaporation of the methanol solution at room temperature.

### Refinement

Non-H atoms were refined anisotropically. H atoms were treated as riding atoms with distances C—H = 0.97 Å (CH_2_) and 0.93 Å (CH). The isotropic displacement parameters for all H atoms were set equal to 1.2 *U*_eq_ of the carrier atom.

### Crystal data

**Table:**

C_31_H_29_N_3_	*F*(000) = 944
*M**_r_* = 443.57	*D*_x_ = 1.215 Mg m^−^^3^
Monoclinic, *P*2_1_/*c*	Melting point = 403–405 K
Hall symbol: -P 2ybc	Mo *K*α radiation, λ = 0.71073 Å
*a* = 8.6623 (6) Å	Cell parameters from 3325 reflections
*b* = 31.582 (2) Å	θ = 2.4–24.3°
*c* = 8.9187 (6) Å	µ = 0.07 mm^−^^1^
β = 96.312 (1)°	*T* = 293 K
*V* = 2425.1 (3) Å^3^	Tabular, yellow
*Z* = 4	0.43 × 0.18 × 0.12 mm

### Data collection

**Table:**

Bruker SMART APEX CCD area-detector diffractometer	4757 independent reflections
Radiation source: fine-focus sealed tube	3374 reflections with *I* > 2σ(*I*)
graphite	*R*_int_ = 0.026
φ and ω scans	θ_max_ = 26.0°, θ_min_ = 2.4°
Absorption correction: multi-scan (*SADABS*; Sheldrick, 1996)	*h* = −10→10
*T*_min_ = 0.970, *T*_max_ = 0.991	*k* = −18→38
13496 measured reflections	*l* = −10→11

### Refinement

**Table:**

Refinement on *F*^2^	Primary atom site location: structure-invariant direct methods
Least-squares matrix: full	Secondary atom site location: difference Fourier map
*R*[*F*^2^ > 2σ(*F*^2^)] = 0.046	Hydrogen site location: inferred from neighbouring sites
*wR*(*F*^2^) = 0.119	H-atom parameters constrained
*S* = 1.01	*w* = 1/[σ^2^(*F*_o_^2^) + (0.0541*P*)^2^ + 0.1994*P*] where *P* = (*F*_o_^2^ + 2*F*_c_^2^)/3
4757 reflections	(Δ/σ)_max_ < 0.001
307 parameters	Δρ_max_ = 0.12 e Å^−^^3^
0 restraints	Δρ_min_ = −0.14 e Å^−^^3^

### Special details

**Table:**

Geometry. All e.s.d.'s (except the e.s.d. in the dihedral angle between two l.s. planes) are estimated using the full covariance matrix. The cell e.s.d.'s are taken into account individually in the estimation of e.s.d.'s in distances, angles and torsion angles; correlations between e.s.d.'s in cell parameters are only used when they are defined by crystal symmetry. An approximate (isotropic) treatment of cell e.s.d.'s is used for estimating e.s.d.'s involving l.s. planes.
Refinement. Refinement of *F*^2^ against ALL reflections. The weighted *R*-factor *wR* and goodness of fit *S* are based on *F*^2^, conventional *R*-factors *R* are based on *F*, with *F* set to zero for negative *F*^2^. The threshold expression of *F*^2^ > σ(*F*^2^) is used only for calculating *R*-factors(gt) *etc*. and is not relevant to the choice of reflections for refinement. *R*-factors based on *F*^2^ are statistically about twice as large as those based on *F*, and *R*- factors based on ALL data will be even larger.

### Fractional atomic coordinates and isotropic or equivalent isotropic displacement parameters (Å^2^)

**Table:**

	*x*	*y*	*z*	*U*_iso_*/*U*_eq_	
C1	−0.02475 (16)	0.43714 (5)	0.59089 (17)	0.0523 (4)	
C2	−0.12646 (18)	0.40495 (6)	0.5389 (2)	0.0653 (4)	
H2	−0.1038	0.3866	0.4627	0.078*	
C3	−0.2618 (2)	0.40148 (7)	0.6050 (2)	0.0791 (5)	
H3	−0.3313	0.3800	0.5737	0.095*	
C4	−0.2981 (2)	0.42893 (7)	0.7167 (2)	0.0820 (6)	
H4	−0.3912	0.4256	0.7583	0.098*	
C5	−0.19874 (19)	0.46114 (6)	0.76719 (19)	0.0693 (5)	
H5	−0.2245	0.4798	0.8412	0.083*	
C6	−0.05852 (17)	0.46530 (5)	0.70515 (16)	0.0539 (4)	
C7	0.07318 (18)	0.49316 (5)	0.73388 (16)	0.0543 (4)	
C8	0.1095 (2)	0.52700 (6)	0.83139 (19)	0.0702 (5)	
H8	0.0393	0.5360	0.8964	0.084*	
C9	0.2502 (3)	0.54688 (7)	0.8303 (2)	0.0851 (6)	
H9	0.2746	0.5697	0.8945	0.102*	
C10	0.3566 (2)	0.53354 (6)	0.7351 (2)	0.0851 (6)	
H10	0.4517	0.5473	0.7376	0.102*	
C11	0.3241 (2)	0.50022 (6)	0.6364 (2)	0.0682 (5)	
H11	0.3956	0.4913	0.5726	0.082*	
C12	0.18125 (17)	0.48054 (5)	0.63596 (16)	0.0524 (4)	
C13	0.19565 (18)	0.42690 (5)	0.42926 (17)	0.0581 (4)	
H13A	0.2482	0.4483	0.3758	0.070*	
H13B	0.1172	0.4139	0.3578	0.070*	
C14	0.31251 (17)	0.39346 (5)	0.48825 (18)	0.0567 (4)	
H14A	0.3657	0.3830	0.4055	0.068*	
H14B	0.3895	0.4062	0.5617	0.068*	
C15	0.23723 (17)	0.35671 (5)	0.56068 (18)	0.0548 (4)	
H15A	0.1568	0.3451	0.4879	0.066*	
H15B	0.1870	0.3674	0.6450	0.066*	
C16	0.34575 (17)	0.32109 (5)	0.61709 (19)	0.0592 (4)	
H16A	0.4312	0.3327	0.6839	0.071*	
H16B	0.3888	0.3083	0.5319	0.071*	
C17	0.26530 (18)	0.28701 (5)	0.70071 (17)	0.0571 (4)	
H17A	0.3424	0.2672	0.7459	0.069*	
H17B	0.2156	0.3000	0.7814	0.069*	
C18	0.14417 (17)	0.26304 (5)	0.59738 (16)	0.0545 (4)	
H18A	0.0780	0.2833	0.5395	0.065*	
H18B	0.1966	0.2464	0.5268	0.065*	
C19	−0.07789 (17)	0.24836 (5)	0.74837 (16)	0.0563 (4)	
C20	−0.1313 (2)	0.28859 (6)	0.7791 (2)	0.0696 (5)	
H20	−0.0829	0.3129	0.7484	0.084*	
C21	−0.2595 (2)	0.29058 (8)	0.8573 (2)	0.0839 (6)	
H21	−0.2985	0.3170	0.8801	0.101*	
C22	−0.3322 (2)	0.25458 (9)	0.9029 (2)	0.0886 (6)	
H22	−0.4174	0.2573	0.9572	0.106*	
C23	−0.2812 (2)	0.21486 (8)	0.8698 (2)	0.0789 (5)	
H23	−0.3316	0.1908	0.8994	0.095*	
C24	−0.15163 (18)	0.21176 (6)	0.79052 (18)	0.0615 (4)	
C25	0.04203 (18)	0.19127 (5)	0.67728 (17)	0.0557 (4)	
C26	0.1556 (2)	0.16414 (5)	0.61244 (17)	0.0583 (4)	
C27	0.1020 (2)	0.12817 (6)	0.5325 (2)	0.0752 (5)	
H27	−0.0039	0.1225	0.5171	0.090*	
C28	0.2057 (3)	0.10106 (6)	0.4763 (2)	0.0905 (6)	
H28	0.1690	0.0772	0.4227	0.109*	
C29	0.3625 (3)	0.10872 (7)	0.4982 (2)	0.0906 (6)	
H29	0.4314	0.0900	0.4601	0.109*	
C30	0.4175 (2)	0.14419 (6)	0.5768 (2)	0.0822 (6)	
H30	0.5236	0.1496	0.5915	0.099*	
C31	0.3140 (2)	0.17165 (5)	0.63358 (19)	0.0675 (5)	
H31	0.3513	0.1955	0.6868	0.081*	
N1	0.12011 (13)	0.44707 (4)	0.54812 (14)	0.0530 (3)	
N2	0.04699 (14)	0.23494 (4)	0.67667 (13)	0.0532 (3)	
N3	−0.07470 (16)	0.17632 (4)	0.74442 (15)	0.0653 (4)	

### Atomic displacement parameters (Å^2^)

**Table:**

	*U*^11^	*U*^22^	*U*^33^	*U*^12^	*U*^13^	*U*^23^
C1	0.0462 (8)	0.0539 (9)	0.0569 (9)	0.0074 (7)	0.0055 (7)	0.0089 (7)
C2	0.0521 (9)	0.0678 (11)	0.0752 (11)	0.0046 (8)	0.0042 (8)	−0.0036 (9)
C3	0.0518 (10)	0.0915 (14)	0.0937 (14)	−0.0075 (10)	0.0067 (10)	−0.0035 (12)
C4	0.0504 (10)	0.1153 (17)	0.0822 (13)	−0.0060 (11)	0.0164 (9)	0.0027 (12)
C5	0.0578 (10)	0.0922 (14)	0.0591 (10)	0.0098 (10)	0.0113 (8)	0.0009 (9)
C6	0.0499 (8)	0.0616 (10)	0.0502 (8)	0.0108 (8)	0.0051 (7)	0.0091 (7)
C7	0.0607 (9)	0.0530 (9)	0.0490 (8)	0.0085 (8)	0.0055 (7)	0.0067 (7)
C8	0.0806 (12)	0.0711 (12)	0.0597 (10)	0.0051 (10)	0.0111 (9)	−0.0055 (9)
C9	0.1004 (15)	0.0742 (13)	0.0810 (13)	−0.0167 (12)	0.0114 (11)	−0.0152 (10)
C10	0.0847 (13)	0.0765 (13)	0.0953 (14)	−0.0245 (11)	0.0153 (11)	−0.0006 (11)
C11	0.0692 (11)	0.0612 (11)	0.0774 (11)	−0.0072 (9)	0.0217 (9)	0.0048 (9)
C12	0.0571 (9)	0.0460 (9)	0.0549 (9)	0.0036 (7)	0.0096 (7)	0.0095 (7)
C13	0.0614 (9)	0.0559 (9)	0.0593 (9)	0.0059 (8)	0.0173 (7)	0.0036 (8)
C14	0.0529 (9)	0.0534 (9)	0.0657 (10)	0.0018 (7)	0.0155 (7)	−0.0051 (8)
C15	0.0470 (8)	0.0543 (9)	0.0633 (9)	0.0027 (7)	0.0069 (7)	0.0011 (8)
C16	0.0483 (9)	0.0563 (9)	0.0718 (10)	−0.0002 (7)	0.0020 (7)	−0.0010 (8)
C17	0.0556 (9)	0.0556 (9)	0.0570 (9)	0.0007 (8)	−0.0072 (7)	0.0042 (8)
C18	0.0614 (9)	0.0514 (9)	0.0492 (8)	−0.0038 (8)	−0.0008 (7)	0.0047 (7)
C19	0.0494 (9)	0.0675 (10)	0.0495 (8)	0.0016 (8)	−0.0055 (7)	0.0027 (8)
C20	0.0620 (11)	0.0741 (12)	0.0698 (11)	0.0063 (9)	−0.0058 (9)	−0.0030 (9)
C21	0.0653 (12)	0.1049 (17)	0.0790 (13)	0.0191 (12)	−0.0024 (10)	−0.0109 (12)
C22	0.0565 (11)	0.132 (2)	0.0766 (13)	0.0119 (13)	0.0043 (9)	0.0004 (13)
C23	0.0577 (11)	0.1061 (16)	0.0713 (11)	−0.0047 (11)	0.0005 (9)	0.0150 (11)
C24	0.0500 (9)	0.0769 (12)	0.0552 (9)	−0.0013 (9)	−0.0045 (7)	0.0091 (8)
C25	0.0596 (9)	0.0544 (9)	0.0509 (8)	−0.0056 (8)	−0.0028 (7)	0.0077 (7)
C26	0.0719 (11)	0.0510 (9)	0.0514 (9)	−0.0028 (8)	0.0036 (8)	0.0098 (7)
C27	0.0954 (13)	0.0573 (11)	0.0721 (11)	−0.0074 (10)	0.0051 (10)	0.0008 (9)
C28	0.130 (2)	0.0652 (13)	0.0764 (13)	0.0002 (14)	0.0136 (13)	−0.0099 (10)
C29	0.1219 (19)	0.0758 (14)	0.0791 (13)	0.0205 (14)	0.0334 (13)	0.0018 (11)
C30	0.0835 (13)	0.0815 (14)	0.0845 (13)	0.0088 (11)	0.0221 (11)	0.0086 (11)
C31	0.0760 (12)	0.0589 (10)	0.0680 (10)	0.0024 (9)	0.0101 (9)	0.0019 (8)
N1	0.0529 (7)	0.0477 (7)	0.0601 (7)	0.0043 (6)	0.0135 (6)	0.0019 (6)
N2	0.0552 (7)	0.0522 (8)	0.0506 (7)	−0.0037 (6)	−0.0017 (6)	0.0033 (6)
N3	0.0630 (8)	0.0658 (9)	0.0659 (8)	−0.0074 (7)	0.0024 (7)	0.0131 (7)

### Geometric parameters (Å, °)

**Table:**

C1—N1	1.3870 (18)	C16—H16A	0.9700
C1—C2	1.391 (2)	C16—H16B	0.9700
C1—C6	1.407 (2)	C17—C18	1.520 (2)
C2—C3	1.373 (2)	C17—H17A	0.9700
C2—H2	0.9300	C17—H17B	0.9700
C3—C4	1.382 (3)	C18—N2	1.4585 (18)
C3—H3	0.9300	C18—H18A	0.9700
C4—C5	1.376 (3)	C18—H18B	0.9700
C4—H4	0.9300	C19—N2	1.3823 (19)
C5—C6	1.395 (2)	C19—C20	1.390 (2)
C5—H5	0.9300	C19—C24	1.392 (2)
C6—C7	1.441 (2)	C20—C21	1.377 (3)
C7—C8	1.392 (2)	C20—H20	0.9300
C7—C12	1.406 (2)	C21—C22	1.383 (3)
C8—C9	1.372 (3)	C21—H21	0.9300
C8—H8	0.9300	C22—C23	1.373 (3)
C9—C10	1.386 (3)	C22—H22	0.9300
C9—H9	0.9300	C23—C24	1.395 (2)
C10—C11	1.380 (3)	C23—H23	0.9300
C10—H10	0.9300	C24—N3	1.388 (2)
C11—C12	1.384 (2)	C25—N3	1.3181 (19)
C11—H11	0.9300	C25—N2	1.3799 (19)
C12—N1	1.3858 (19)	C25—C26	1.470 (2)
C13—N1	1.4527 (18)	C26—C31	1.384 (2)
C13—C14	1.517 (2)	C26—C27	1.393 (2)
C13—H13A	0.9700	C27—C28	1.375 (3)
C13—H13B	0.9700	C27—H27	0.9300
C14—C15	1.511 (2)	C28—C29	1.372 (3)
C14—H14A	0.9700	C28—H28	0.9300
C14—H14B	0.9700	C29—C30	1.378 (3)
C15—C16	1.516 (2)	C29—H29	0.9300
C15—H15A	0.9700	C30—C31	1.383 (2)
C15—H15B	0.9700	C30—H30	0.9300
C16—C17	1.521 (2)	C31—H31	0.9300
			
N1—C1—C2	129.25 (14)	H16A—C16—H16B	107.8
N1—C1—C6	108.82 (13)	C18—C17—C16	112.21 (12)
C2—C1—C6	121.92 (14)	C18—C17—H17A	109.2
C3—C2—C1	117.02 (17)	C16—C17—H17A	109.2
C3—C2—H2	121.5	C18—C17—H17B	109.2
C1—C2—H2	121.5	C16—C17—H17B	109.2
C2—C3—C4	122.15 (18)	H17A—C17—H17B	107.9
C2—C3—H3	118.9	N2—C18—C17	113.94 (12)
C4—C3—H3	118.9	N2—C18—H18A	108.8
C5—C4—C3	121.04 (16)	C17—C18—H18A	108.8
C5—C4—H4	119.5	N2—C18—H18B	108.8
C3—C4—H4	119.5	C17—C18—H18B	108.8
C4—C5—C6	118.71 (16)	H18A—C18—H18B	107.7
C4—C5—H5	120.6	N2—C19—C20	131.73 (16)
C6—C5—H5	120.6	N2—C19—C24	106.03 (14)
C5—C6—C1	119.14 (15)	C20—C19—C24	122.25 (16)
C5—C6—C7	133.92 (15)	C21—C20—C19	116.48 (19)
C1—C6—C7	106.93 (12)	C21—C20—H20	121.8
C8—C7—C12	119.05 (15)	C19—C20—H20	121.8
C8—C7—C6	134.42 (15)	C20—C21—C22	122.1 (2)
C12—C7—C6	106.53 (13)	C20—C21—H21	119.0
C9—C8—C7	119.13 (17)	C22—C21—H21	119.0
C9—C8—H8	120.4	C23—C22—C21	121.35 (19)
C7—C8—H8	120.4	C23—C22—H22	119.3
C8—C9—C10	121.08 (18)	C21—C22—H22	119.3
C8—C9—H9	119.5	C22—C23—C24	118.0 (2)
C10—C9—H9	119.5	C22—C23—H23	121.0
C11—C10—C9	121.31 (18)	C24—C23—H23	121.0
C11—C10—H10	119.3	N3—C24—C19	109.88 (14)
C9—C10—H10	119.3	N3—C24—C23	130.28 (17)
C10—C11—C12	117.63 (17)	C19—C24—C23	119.83 (18)
C10—C11—H11	121.2	N3—C25—N2	112.69 (14)
C12—C11—H11	121.2	N3—C25—C26	123.34 (15)
C11—C12—N1	129.06 (14)	N2—C25—C26	123.96 (13)
C11—C12—C7	121.78 (15)	C31—C26—C27	118.61 (17)
N1—C12—C7	109.16 (13)	C31—C26—C25	122.84 (15)
N1—C13—C14	112.91 (12)	C27—C26—C25	118.47 (16)
N1—C13—H13A	109.0	C28—C27—C26	119.99 (19)
C14—C13—H13A	109.0	C28—C27—H27	120.0
N1—C13—H13B	109.0	C26—C27—H27	120.0
C14—C13—H13B	109.0	C29—C28—C27	121.0 (2)
H13A—C13—H13B	107.8	C29—C28—H28	119.5
C15—C14—C13	112.35 (12)	C27—C28—H28	119.5
C15—C14—H14A	109.1	C28—C29—C30	119.8 (2)
C13—C14—H14A	109.1	C28—C29—H29	120.1
C15—C14—H14B	109.1	C30—C29—H29	120.1
C13—C14—H14B	109.1	C29—C30—C31	119.6 (2)
H14A—C14—H14B	107.9	C29—C30—H30	120.2
C14—C15—C16	115.52 (12)	C31—C30—H30	120.2
C14—C15—H15A	108.4	C30—C31—C26	121.00 (18)
C16—C15—H15A	108.4	C30—C31—H31	119.5
C14—C15—H15B	108.4	C26—C31—H31	119.5
C16—C15—H15B	108.4	C12—N1—C1	108.55 (12)
H15A—C15—H15B	107.5	C12—N1—C13	124.91 (12)
C15—C16—C17	112.76 (12)	C1—N1—C13	126.53 (13)
C15—C16—H16A	109.0	C25—N2—C19	106.15 (13)
C17—C16—H16A	109.0	C25—N2—C18	129.05 (13)
C15—C16—H16B	109.0	C19—N2—C18	124.10 (13)
C17—C16—H16B	109.0	C25—N3—C24	105.24 (14)

### Hydrogen-bond geometry (Å, °)

**Table:**

*D*—H···*A*	*D*—H	H···*A*	*D*···*A*	*D*—H···*A*
C28—H28···Cg1	0.93	2.78	3.665 (2)	159
C18—H18A···Cg2	0.97	2.87	3.596 (3)	133

## References

[bb1] Allen, F. H., Kennard, O., Watson, D. G., Brammer, L., Orpen, A. G. & Taylor, R. (1987). *J. Chem. Soc. Perkin Trans. 2*, pp. S1–19.

[bb2] Bouwman, E., Driessen, W. L. & Reedijk, J. (1990). *Coord. Chem. Rev.* **104**, 143–172.

[bb3] Huang, W. S., Lin, J. T., Chien, C. H., Tao, Y. T., Sun, S. S. & Wen, Y. S. (2004). *Chem. Mater.* **16**, 2480–2488.

[bb4] Sheldrick, G. M. (1996). *SADABS*. University of Göttingen, Germany.

[bb5] Sheldrick, G. M. (2008). *Acta Cryst.* A**64**, 112–122. 10.1107/S0108767307043930 18156677

[bb6] Si, Z. J., Li, J., Li, B., Zhao, F. F., Liu, S. Y. & Li, W. L. (2007). *Inorg. Chem.* **46**, 6155–6163. 10.1021/ic061645o 17583336

[bb7] Siemens (1996). *SMART* and *SAINT*. Siemens Analytical X-ray Instruments Inc., Madison, Wisconsin, USA.

[bb8] Spasov, A. A., Yozhitsa, I. N., Bugaeva, L. I. & Anisimova, V. A. (1999). *Pharm. Chem. J.* **33**, 232–243.

[bb9] Zhu, Z., Lippa, B., Drach, J. C. & Townsend, L. B. (2000). *J. Med. Chem.* **43**, 2430–2437. 10.1021/jm990290y 10882370

